# Supplementary material for: The genome sequence of a drosophilid fruit fly, Drosophila histrio (Meigen, 1830)
Source: Wellcome Open Res. 2024 Feb 19;9:56. [Version 1] doi: 10.12688/wellcomeopenres.20631.1 (PMC11249525; doi:10.12688/wellcomeopenres.20631.1)
Supplement: Peer review history

# Reviewer report: Reviewer response for version 1

**DOI:** 10.21956/wellcomeopenres.22834.r86348

**Published:** 2024-07-23

**Authors:** Elizabeth Everman

**Affiliations:** 1 School of Biological Sciences, The University of Oklahoma, Norman, Oklahoma, USA

- Figure 3 could be improved by rotating the y axis labels 180 degrees. The legend is overlapping part of the upper panel and the color differences are not very effective because the lower left panel has differences in alpha level which correspond with the figure legend. Are all of the color-coded categories visible? It is not clear to me that anything is gained with the interactive version of the plot.
- Figure 4 could be improved by rotating the y axis label 180 degrees.
- More details regarding library preparation would be helpful, for example referencing a kit or protocol.
- Details in the analytical pipeline should be included, ideally as a supplement with annotated code. If default flags/options were used in the steps, this would be a simple way to add clarity, but if specific flags/options were used these should be noted.

Are sufficient details of methods and materials provided to allow replication by others?

Partly

Is the rationale for creating the dataset(s) clearly described?

Yes

Are the datasets clearly presented in a useable and accessible format?

Yes

Are the protocols appropriate and is the work technically sound?

Yes

Reviewer Expertise:

Quantitative and evolutionary genetics, transcriptomics, genomics

I confirm that I have read this submission and believe that I have an appropriate level of expertise to confirm that it is of an acceptable scientific standard.

# Reviewer report: Reviewer response for version 1

**DOI:** 10.21956/wellcomeopenres.22834.r86351

**Published:** 2024-07-15

**Authors:** Anthony Bayega

**Affiliations:** 1 McGill Genome Centre, Department of Human Genetics, McGill University, Montreal, Québec, Canada

Obbard *et al*. provide an overview description of the genome of *Drosophila histrio* which they sequenced and assembled. I commend them for this. My comments follow:

1. In the background, the authors provide a brief description of the organism and its geographical distribution. They also provide a rationale for sequencing the organism. If the space allows it, it would have helped the reader to know previous efforts in characterizing this organism and anything known prior, such as previous estimation of genome size, number of genes, among others. This is a minor comment as the background is ample in its current format.
2. In the genome report, the authors could consider using genomescope to estimate heterozygosity (since they are using a wild strain) and genome size. It would be interesting to see if the final assembly would be within predicted size from Genomescope. This is a minor comment since their k-mer completeness already showed 100%.

Overall, the authors provide a high quality genome and also assign much of it to chromosomes. Although much work remains to order the scaffolds and complete the gaps and also structurally and functionally annotate the genome, the current work will indeed be valuable to the whole community. I therefore recommend the publication of this genome so that this resource become widely accessible to the scientific community.

Are sufficient details of methods and materials provided to allow replication by others?

Yes

Is the rationale for creating the dataset(s) clearly described?

Yes

Are the datasets clearly presented in a useable and accessible format?

Yes

Are the protocols appropriate and is the work technically sound?

Yes

Reviewer Expertise:

Molecular biology and advanced genomics

I confirm that I have read this submission and believe that I have an appropriate level of expertise to confirm that it is of an acceptable scientific standard.

# Reviewer report: Reviewer response for version 1

**DOI:** 10.21956/wellcomeopenres.22834.r85236

**Published:** 2024-06-20

**Authors:** Gen Morinaga

**Affiliations:** 1 University of Calgary, Calgary, Alberta, Canada

In the article titled “The genome sequence of a drosophilid fruit fly, *Drosophila histrio *(Meigen, 1830)”, Obbard et al., combine PacBio HiFi and HiC sequencing data to assemble a chromosome-scale genome of the fruit fly *Drosophila histrio.* Their protocol is adequate (if a bit brief), and the resulting assembly is highly complete and contiguous. If I had to criticize anything about the paper as it is, it would be that the bioinformatic protocol is very brief—I generally prefer getting more details from methods sections (e.g., flags used for each program). Aside from this (very) minor criticism, I believe the work presented is a valuable addition to the growing library of high-quality invertebrate genomes and should prove useful to those studying *Drosophila* or Diptera biology.

Are sufficient details of methods and materials provided to allow replication by others?

Partly

Is the rationale for creating the dataset(s) clearly described?

Yes

Are the datasets clearly presented in a useable and accessible format?

Yes

Are the protocols appropriate and is the work technically sound?

Yes

Reviewer Expertise:

Genomics, bioinformatics, evolutionary biology

I confirm that I have read this submission and believe that I have an appropriate level of expertise to confirm that it is of an acceptable scientific standard.
